# Geographical differences in osteoporosis, obesity, and sarcopenia related traits in white American cohorts

**DOI:** 10.1038/s41598-019-48734-9

**Published:** 2019-08-23

**Authors:** Yu Zhou, Kehao Wu, Hui Shen, Jigang Zhang, Hong-Wen Deng, Lan-Juan Zhao

**Affiliations:** 1 Center for Bioinformatics and Genomics, Department of Global Biostatistics and Data Science, School of Public Health and Tropical Medicine, Tulane University, New Orleans, LA 70112 USA; 2 Department of Cell and Molecular Biology, Tulane University, New Orleans, LA 70112 USA

**Keywords:** Population screening, Risk factors

## Abstract

It has been reported that geographical variation influences bone mineral density (BMD), obesity, and sarcopenia related traits in other countries. However, there is lack of similar studies in the US population. In this study, we compared data from three US study cohorts to evaluate geographical variations of BMD and body composition. BMD, fat mass and lean mass were collected from Dual-energy X-ray absorptiometry machine. ANCOVA and Chi-square tests were used to compare the differences between BMDs, obesity and sarcopenia related traits from different regional sites (Omaha, Kansas City and Baton Rouge/New Orleans). Eta-squared was used to measure the effect sizes of these differences. A total of 11,315 Caucasians from our previous three study cohorts were compared. There was no significant geographical difference in BMD for males or females under the criteria of p-values < 0.05 and effect size η^2^ > 0.01. There were significant geographical differences with medium effect size (p-value < 0.001, 0.01 < η^2^ < 0.14) for whole body fat mass percentage and index of low muscle mass. For Caucasians in the United States, there is no significant geographical effect found on BMD. The obesity and sarcopenia related traits are significantly different between the three study cohorts.

## Introduction

Osteoporosis, obesity, and sarcopenia (age-related decrease in skeletal muscle mass) are three of the most common health issues in the world. They are influenced by multiple factors, such as age, genetics, gender, and race, but few studies have focused on the potential contributions of geographical location. Geographical variation is indeed associated with a number of important factors for human health, including differences in latitude, sun exposure time, and diet. All these factors are found to be associated with vitamin D level^1–3^ and vitamin D deficiency will result in osteoporosis and sarcopenia^4–6^. Meanwhile, gut bacteria that are known associated with obesity was found to vary by geographic latitude^7^.

Osteoporosis is a skeletal disease characterized by a reduction of bone mineral dentistry (BMD). Dual x-ray absorptiometry (DXA) is widely used to determine BMD in osteoporosis-related studies^8^. Several studies in Europe^9–12^, Canada^13^ and Mexico^14^ have consistently found geographic variation in BMD; however, evidence is scarce in the United States. Only one study^15^ two decades ago showed that there was a small difference (1–4%) in femur BMD between various United States regions. As years passed and the recent rapid development of the study diseases with aging populations, an up-to-date survey is required in the United States.

Obesity is a condition of excessive body fat that causes or exacerbates many public health problems. Studies on geographical variation of obesity are based on body mass index (BMI). For example, using BMI as obesity index, DNPPAO (Division of Nutrition, Physical Activity, and Obesity)^16^ found that there were geographical variations in obesity prevalence in United States. BMI is calculated as total body weight divided by the square of the body height. Body weight is a heterogeneous phenotype, consisting of fat mass, muscle mass, and bone mass. There are several alternative techniques to BMI to define obesity^17^, such as waist circumference, skinfold thickness, whole body fat mass percentage (WBFP) and *etc*. Rather than BMI, WBFP would better identify metabolic risk^18^ and by DXA, WBFP could be accurately determined comparing to other alternative techniques^17^. To the best of our knowledge, no studies have evaluated geographical variations of obesity using WBFP in United States.

Sarcopenia is a condition characterized by age-related loss of skeletal muscle mass and strength^19^. Individuals with sarcopenia exhibited impaired balance, increased physical disability, poor quality of life, and death^20^. Despite there is no universally accepted operational definition of sarcopenia at this time^21,22^, the recent announcement of an ICD-10 code (M62.84) dedicated to sarcopenia is an important step towards recognizing sarcopenia as an independent disease entity and increasing clinical awareness of the condition^23^. Most of the proposed operational definitions for sarcopenia^24–28^ included appendicular lean mass (ALM), which is the sum of the lean mass of both arms and legs measured by DXA, as an approximation of muscle mass, and recommended using the ratio of ALM over height squared (ALM/ht^2^) as an index of low muscle mass (ALMI). A project conducted by multiple centers in Europe and US showed that there were differences in mean ALM and ALMI among the subjects from the studies performed in different regions^28^. However, the races of subjects in this project were various. To the best of our knowledge, there are no comprehensive studies on geographical variations in ALMI in American Caucasians.

The objective of our study was to determine whether there are geographical variations of BMD and body composition in Caucasians. We compared BMD, WBFP, and ALMI for Caucasian subjects from three different regional studies in United States.

## Results

### Basic characteristics

Table 1 shows the basic characteristics and lifestyle information of the study subjects, which was divided into six subgroups by geographical location and gender. There was a total of 11,315 subjects (Age: 52.1 ± 14.9 years, 7,076 females, and 4,239 males). For both genders, significant differences in age, height and weight were observed among the three study cohorts (p-value < 0.001). Meanwhile, lifestyle was significantly different among the subjects from the three geographical regions. For example, compared with LOS and OOS, the subjects from KCOS had largest percentage of subjects with regular physical exercise and smallest percentage for alcohol use, but the percentage of smokers for men and women was largest and second largest, respectively.

**Table 1:** Basic characteristic of subjects from LOS, KCOS and OOS. (mean ± SD).

Gender	Traits	LOS^a^	KCOS^a^	OOS^a^	P-value	
Female	Sample Size (number)	3,977	2,066	1,033		
	Age (years)	52.0 ± 15.3	47.7 ± 13.2	63.3 ± 9.5	<0.001	KCOS < LOS < OOS
	Height (cm)	162.7 ± 6.4	163.4 ± 6.4	162.1 ± 6.3	<0.001	OOS < LOS < KCOS
	Weight (kg)	70.6 ± 16.8	71.9 ± 16.7	72.6 ± 15.3	<0.001	LOS < KCOS < OOS
	Exercise (%)^b^	69.7 (68.2–71.0)	78.2 (76.5–80.0)	61.6 (57.2–65.9)	<0.001	OOS < LOS < KCOS
	Alcohol (%)^c^	79.2 (78.0–80.5)	54.2 (51.1–57.3)	66.6 (61.4–71.7)	<0.001	KCOS < OOS < LOS
	Smoke (%)^d^	39.5 (38.0–41.0)	39.1 (37.0–41.2)	23.7 (20.8–26.6)	<0.001	OOS < KCOS < LOS
Male	Sample Size (number)	1,962	1,431	846		
	Age (years)	49.2 ± 15.4	42.2 ± 12.9	64.3 ± 9.6	<0.001	KCOS < LOS < OOS
	Height (cm)	175.5 ± 6.9	175.9 ± 6.8	176.3 ± 6.7	<0.001	LOS < KCOS < OOS
	Weight (kg)	84.0 ± 16.4	83.7 ± 16.9	90.5 ± 14.9	<0.001	KCOS < LOS < OOS
	Exercise (%)	73.9 (72.0–75.9)	84.6 (82.8–86.5)	59.8 (55.0–64.7)	<0.001	OOS < LOS < KCOS
	Alcohol (%)	78.7 (76.9–80.5)	62.1 (59.3–65.0)	75.7 (70.1–80.7)	<0.001	KCOS < OOS < LOS
	Smoke (%)	64.9 (62.8–67.1)	77.3 (75.2–79.5)	39.4 (35.7–43.1)	<0.001	OOS < LOS < KCOS

Note:

^a^LOS: Louisiana Osteoporosis Study; KCOS: Kansas City Osteoporosis Study; OOS: Omaha Osteoporosis Study.

^b^Exercise is the abbreviation for percentage of subjects who self-reported regular exercise. 95% confidence interval of percentage were reported in the bracket.

^c^Alcohol is the abbreviation for percentage of subjects who self-reported alcohol use. 95% confidence interval of percentage were reported in the bracket.

^d^Smoke is the abbreviation for percentage of subjects who self-reported smoking. 95% confidence interval of percentage were reported in the bracket.

### Geographical differences of BMD

Table 2 shows the geographical differences of BMD among LOS, KCOS, and OOS cohorts. HIP-BMD, FNK-BMD, and SPN-BMD were compared by the ANCOVA model, controlling for age, height, weight, smoking, alcohol use, and regular physical activity. FNK-BMD and SPN-BMD were not significantly different between genders among the three studies under the criteria of p < 0.05, whereas there were geographical differences for HIP-BMD (p = 0.004 for females and p = 0.003 for males). However, due to the limitations of a null hypothesis test, when a large sample size is used, even a trivial effect could be exaggerated with small p-values^29,30^. In the study, the effect sizes (eta-squared) were reported to determine the regional differences. When HIP-BMD values were compared among the three cohorts, the effect size was smaller than 0.01 (Table 2), which is considered to be the cutoff value of small effect size^31^. The results indicate no significant geographical difference in BMD for males or females under the criteria of η^2^ > 0.01.

**Table 2:** BMD of subjects from LOS, KCOS and OOS (mean ± SD).

Gender	Traits	LOS	KCOS	OOS	P^a^	η^2b^
Female	Sample Size	3,977	2,066	1,033		
	HIP (g/cm^2^)	0.92 ± 0.14	0.95 ± 0.14	0.87 ± 0.15	<0.01	0.001
	FNK (g/cm^2^)	0.75 ± 0.13	0.79 ± 0.13	0.72 ± 0.13	NS	0.001
	SPN (g/cm^2^)	0.99 ± 0.15	1.02 ± 0.14	0.96 ± 0.17	NS	<0.001
Male	Sample Size	1,962	1,431	846		
	HIP (g/cm^2^)	1.01 ± 0.14	1.02 ± 0.14	1.01 ± 0.14	<0.01	0.002
	FNK (g/cm^2^)	0.82 ± 0.14	0.86 ± 0.14	0.8 ± 0.13	NS	0.001
	SPN (g/cm^2^)	1.05 ± 0.16	1.06 ± 0.14	1.07 ± 0.18	NS	<0.001

Note:

^a^NS stands for not significant, p > 0.05.

^b^η^2^ is eta-squared effect size of ANOVA. In the study, we consider an effect size larger than 0.01 as significant.

### Geographical differences in obesity and sarcopenia traits

For all three body composition traits (BMI, WBFP, and ALMI), we identified significant geographical differences (p-values < 0.001) among the three datasets (LOS, KCOS and OOS), even when adjusted for age, height, weight, smoking, alcohol use, and regular physical activity (Table 3). The geographical patterns for BMI were the same for both female and male subjects (KCOS < LOS < OOS). Although BMI showed significant differences among three groups (p-values < 0.001), the effect size was relatively small (η^2^ = 0.004 for female and η^2^ = 0.003 for male). WBFP showed the same ranking pattern in all three study regions (KCOS < LOS < OOS) as BMI. For sarcopenia, subjects from KCOS had the highest ALMI value and subjects from LOS had the lowest ALMI value. The effect size of geographical differences was large for WBFP and ALMI (η^2^ > 0.01).

**Table 3:** Body composition of subjects from LOS, KCOS and OOS (mean ± SD).

Gender	Traits	LOS	KCOS	OOS	P	η^2^	
Female	Sample Size	3,977	2,066	1,033			
	BMI (kg/m^2^)	26.7 ± 6.17	26.9 ± 6.2	27.6 ± 5.6	<0.001	0.004	LOS < KCOS < OOS
	WBFP (%)	36.8 ± 6.6	32.4 ± 7.1	38.2 ± 6.0	<0.001	**0**.**096**	KCOS < LOS < OOS
	ALMI (kg/m^2^)	6.9 ± 1.1	7.7 ± 1.1	7.1 ± 1.1	<0.001	**0**.**045**	LOS < OOS < KCOS
Male	Sample Size	1,962	1,431	846			
	BMI (kg/m^2^)	27.2 ± 4.9	27.0 ± 4.9	29.0 ± 4.3	<0.001	0.003	KCOS < LOS < OOS
	WBFP (%)	25.6 ± 5.7	20.4 ± 6.5	27.5 ± 5.5	<0.001	**0**.**102**	KCOS < LOS < OOS
	ALMI (kg/m^2^)	8.8 ± 1.2	9.6 ± 1.2	9.2 ± 1.1	<0.001	**0**.**033**	LOS < OOS < KCOS

Note:

η^2^ is eta-squared effect size of ANOVA. In the study, we consider an effect size bigger than 0.01 as significant.

**Figure 1:**
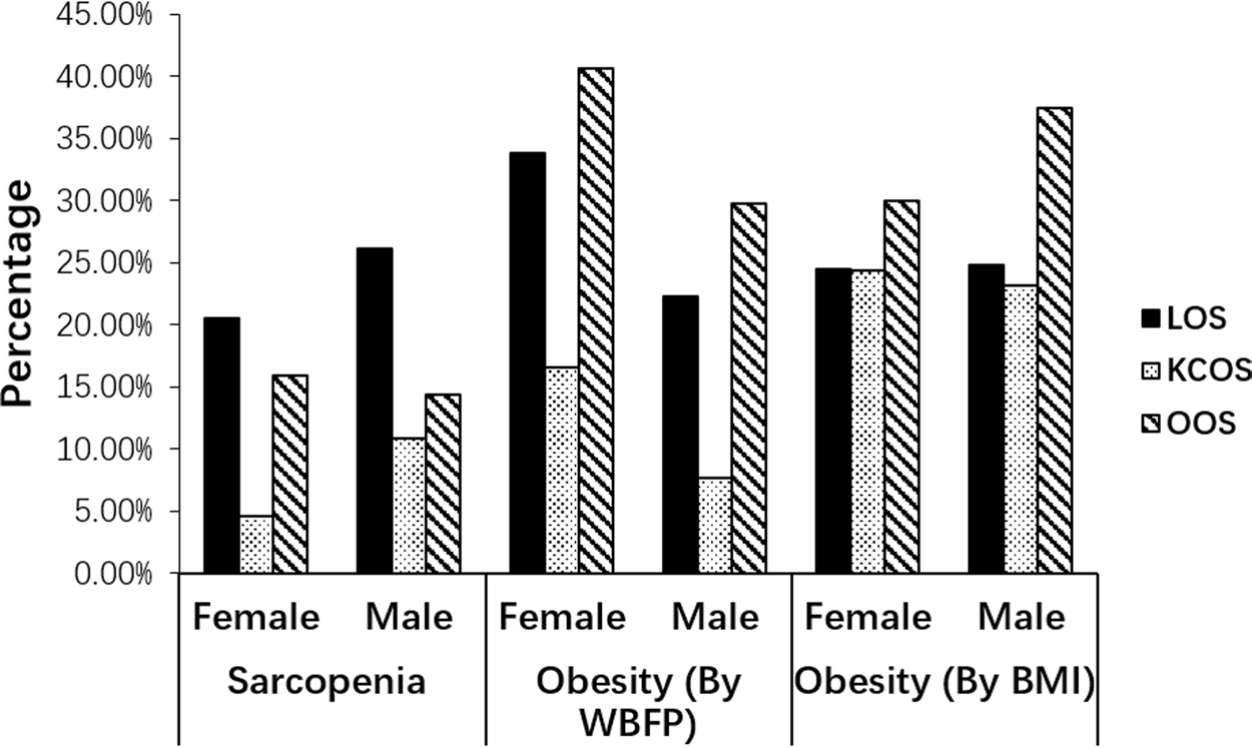
Prevalence of sarcopenia and obesity in subjects from LOS, KCOS, and OOS. The sex-specific cutoff values for sarcopenia are 7.26 kg/m^2^ (male) and 5.45 kg/m^2^ (female). Using BMI value, obesity was defined as BMI ≥ 30 kg/m^2^. Using WBFP, the cutoff points of 30% and 40% were proposed for males and females, respectively.

Figure 1 shows the prevalence of sarcopenia and obesity in the three datasets. The sex-specific cutoff values for sarcopenia defined by ALMI were 7.26 kg/m^2^ (male) and 5.45 kg/m^2^ (female). The cutoff values for obesity defined by WBFP were 30% (male) and 40% (female). For subjects from KCOS, both males and females had the lowest rates of sarcopenia (4.6% for females and 10.94% for males) and obesity defined by WBFP threshold (16.61% for females and 7.73% for males). Subjects from OOS had the highest percentage of obesity in both male and female subjects, regardless of whether it is defined by WBFP or BMI. When obesity was defined by WBFP, significant differences can be found in each pair of datasets; however, when obesity was defined as BMI ≥ 30 kg/m^2^, there were no significant differences in prevalence of obesity for subjects between KCOS and LOS (female: 24.37% vs 24.51% and male: 23.13% vs 24.81%). This indicates that it is easier to identify dataset differences using WBFP than using BMI.

## Discussion

The purpose of this study was to determine whether there were geographical differences in BMD and body composition of non-Hispanic whites in the United States. After adjusting for age, weight, height, smoking, alcohol usage, and regular physical activity, we found no significant differences among the studies from three geographical regions for HIP-BMD, FNK-BMD, and SPN-BMD, if we use stringent criteria p-values < 0.05 and η^2^ > 0.01. Significant geographical differences were found for body composition phenotypes, such as WBFP and ALMI.

To the best of our knowledge, in the past decade, this is the only study to compare the geographical differences of both BMD and body composition of non-Hispanic whites in the United States. Our data showed that there was no regional effect on BMD variation in three selected geographical regions in the United States. This is different from studies in other countries. In previous studies, European between-center differences in BMD were observed in the European Vertebral Osteoporosis (EVO)^9^, the Network in Europe on Male Osteoporosis (NEMO)^10^ and the Osteoporosis and Ultrasound study (OPUS)^12^. In a study published in 1997^9^ of 16 European populations, both males and females aged between 50 and 80 were recruited randomly from local population registers. BMD values were determined by DXA from Hologic, Lunar, or Norland^9^. The study demonstrated that there are major differences between BMD values in European population samples from different geographical sites^9^. In another study conducted in 2008^10^, normal hip and spine BMD data of 1,163 males and 329 females aged 19–29 were collected from 17 centers across Europe with strong evidence of heterogeneity among countries, but not among centers of the same country. In 2009^11^, 10,504 hip BMD scans conducted during 1997–2002 from two population-based studies across Norway were used to validate the geographical difference. The authors found that women ≥60 years in Tromsø had higher age-adjusted BMD than women in Bergen, whereas BMD among women <60 years was similar in Tromsø and Bergen. For men, age-adjusted total hip BMD was lower in Bergen compared with Tromsø. In 2011^12^, proximal femur BMD of females aged 20–39 (n = 258) and males aged 55–79 (n = 1,426) from three European centers were compared. A clear geographical difference in hip BMD did exist; however, there was less evidence for femoral neck BMD^12^. Similar research was also conducted in Canada^13^ and Mexico^14^ and found geographical variation in BMD. Older survey results^15^ (earlier than 2000) showed that although there were differences among regions in the United States (BMD levels of whites tended to be lower in the southern United States), the magnitude of this difference was small. They found that mean total femur BMD levels in the North, Central and West regions were only approximately 0.02–0.03 g/cm^2^, or 1–4%, higher than in the South. In our study, we did not find significant differences in FNK-BMD or SPN-BMD among the three groups. For HIP-BMD, although the p-value was less than 0.05, the effect size was smaller than 0.01. This indicates that the geographic differences had minimal effects on HIP-BMD variation. These controversial results can be explained by the adjustment of lifestyle factors in our study, which include smoking, exercise, and alcohol use. These factors were significantly different among the cohorts and may affect BMD variation^32–34^.

In this study, we found that geographical locations were associated with WBFP and ALMI, which is used to investigate obesity and sarcopenia, respectively. Our WBFP results were consistent with a previous meta-analysis of literature data from global studies^35^. It showed that European Caucasians had higher (P < 0.05) body fat percentage (28.4%) compared to the American Caucasians (23.4%)^35^. For sarcopenia, a large project using pooled sample (n = 26,625 participants) showed that, for both genders, there were differences in mean ALM and ALMI among the subjects from the studies performed in different regions^28^, including multiple regions in USA and Europe. However, this project did not conduct a detailed analysis of geographical variations in ALM or ALMI, such as dividing the subjects into various geographical areas for further comparison, and also contained a small number of non-white subjects^28^. In our study, only Caucasian subjects were recruited and ALM and ALMI were also found to vary by different geographic locations.

Different definitions of obesity may lead to different results. If we use BMI as a phenotype to define obesity in the study, there was no significant difference in the prevalence of obesity for subjects from LOS and KCOS (Fig. 1). If we use WBFP as criteria for obesity classification, the obesity rate of KCOS was dramatically reduced from 23.13% (by BMI) to 7.73% (by WBFP) for males and reduced from 24.37% (by BMI) to 16.61% (by WBFP) for females. For LOS, the percentage of obesity among female subjects increased from 24.51% (by BMI) to 33.83% (by WBFP) and the obesity rate of male subjects decreased slightly, from 24.81% (by BMI) to 22.24% (by WBFP). These results indicate that BMI should not be the only standard for obesity, especially when study geographical difference which involve subjects from different regions. A systematic review and meta-analysis^36^ found that commonly used BMI cutoff values have high specificity but low sensitivity to identify excessive body adiposity. Thus, WBFP may be a good additional measurement to identify geographical difference for traits related to obesity.

Our study has several advantages compared to previous studies. First, in this study, lifestyle factors (exercise, smoking and alcohol use) were controlled to evaluate the effect of geographical variation on BMD and body composition. Second, the three cohorts were recruited by the same research group using the same inclusion and exclusion criteria. This may reduce the selection bias. Third, we used the same standard bone osteodensitometers machine, QDR 4500, the gold standard for bone mass measurement in humans^37^, which may further reduce systematic errors. Fourth, in this study, effect size was considered in order to show the degree of difference. In previous studies, p-value was the only way that data were compared. A proper inference requires full reporting as suggested by SAS in 2016^38^. P-value does not measure the size of an effect or the importance of a result.

Our study had some limitations. First, our sample size was relatively small. Only three regions were included in the study. We did not recruit subjects from western or eastern parts of the United States. Second, we could not investigate the factors that may cause the regional variation of BMD and body composition, like diet or sun exposure. Since we found WBFP variation among the three locations in different latitudes, for the next step, we would like to conduct further studies and try to define if the WBFP variation was caused by the potential mechanisms, like different sun exposure time via vitamin D metabolism, temperature variation in different latitudes or diet differences.

In summary, we found geographical differences of obesity and sarcopenia when defined by WBFP and ALMI respectively. No significant geographical effects on BMD variation were identified. The regional effects on obesity and sarcopenia were still significant after being adjusted for age, weight, height, smoking, alcohol use, and regular physical activity. This indicates that unknown region-related factors may contribute to variations in body composition. We also found that the geographical differences in obesity were easier to identify using WBFP, compared with BMI.

## Materials and Methods

All the methods were conducted in accordance with the rules and guidelines of the Institutional Review Boards of University of Missouri Kansas City, Creighton University and Tulane University. The Institutional Review Boards of University of Missouri Kansas City, Creighton University and Tulane University approved the study. Written informed consent was obtained from all participants before inclusion in the study.

### Study participants

This study used 11,315 randomly recruited Caucasian subjects, from three cross-sectional study cohorts: 5,939 from the Louisiana Osteoporosis Study (LOS, New Orleans and Baton Rouge area, Coordinates: ~30^o^N~90–91^o^W)^39^, 3,497 from the Kansas City Osteoporosis Study (KCOS, Kansas City area, Coordinates: ~39^o^N~94^o^W)^40^, and 1,879 from the Omaha Osteoporosis Study (OOS, Omaha area, Coordinates: ~41^o^N~96^o^W)^41^. All samples were approved by the respective institutional ethics review boards and all participants signed informed-consent documents before entering the studies. Only healthy people (defined by the exclusion criteria) were included in the analyses. All of the study subjects were Caucasians with age > 18 years. The exclusion criteria are:

1. Female subjects who are or could be pregnant;
2. Female subjects who have had bilateral oophorectomy;
3. Serious residuals from cerebral vascular disease;
4. Diabetes mellitus, except for those controlled under medication;
5. Chronic renal failure;
6. Chronic liver failure;
7. Significant chronic lung disease;
8. Alcohol abuse as defined by those who cannot limit drinking, get drunk regularly, and cannot fulfill major responsibilities at work, school, or home.
9. Chronic obstructive pulmonary disease (COPD);
10. Corticosteroid therapy at pharmacologic levels for more than 6 months duration;
11. Treatment with anticonvulsant therapy for more than 6 months duration;
12. Evidence of other metabolic or inherited bone disease such as hyper- or hypoparathyroidism, Paget’s disease, osteomalacia, osteogenisis imperfecta or others;
13. Rheumatoid arthritis (except for minor cases that involve only hand joint and wrist);
14. Collagen disease (i.e., osteogenesis imperfecta and hypochondrogenesis);
15. Chronic gastrointestinal diseases including celiac disease, postgastrectomy, Crohn’s disease, ulcerative colitis, liver transplant, cirrhosis;
16. Weight over 300 pounds.

A medical history questionnaire was given to each subject to obtain self-reported information on age, sex, family history, physical activity, drinking, and smoking history.

### Measurement of BMD and body composition

All BMDs at regional sites, including hip (HIP-BMD), spine (SPN-BMD), femoral neck (FNK-BMD) were measured, as were body composition including fat mass and lean mass at all regional sites, using Hologic QDR-4500 DXA scanners. DXA instruments were operated by trained technicians and calibrated daily by measuring spine phantoms. Long-term precision in our hands, the coefficient of variation (CV), was less than 1.6% for BMD measurement.

WBFP was calculated as whole body fat mass divided by whole body mass. ALM was determined by the sum of lean mass of both arms and legs (in unit of kilograms). The ALMI was calculated as ALM/height^2^ (in unit of kilograms/meter^2^). BMI was calculated as weight in kilograms divided by the square of height in meters.

### Definition of sarcopenia and obesity

Sarcopenia was defined here using ALMI^25^. If a subject’s ALMI fell into lower than 2 SD below mean of young adults, the subject would be considered as sarcopenia in the study^24,25^. In this definition, sarcopenia was significantly associated with physical disability in both genders, and was independent of ethnicity, age, morbidity, obesity, income and health behaviors^24^. The cutoff values for definition of sarcopenia were ALMI ≤ 7.26 kg/m^2^ for males and ≤ 5.45 kg/m^2^ for females. These cutoff values were broadly accepted in a number of sarcopenia studies (e.g.^24,25,42^).

We defined obesity using BMI or WBFP. The WHO defines obesity as BMI ≥ 30 kg/m^2^. When we used WBFP to define obesity, cutoff values of 30% and 40% were proposed to define obesity for males and females, respectively. These cutoff values were adopted in a wide range of multiple studies (e.g.^43,44^).

### Data and statistical analyses

We performed a Grubbs’ test to remove outliers. We applied a one-tailed test to detect outliers, which have values statistically different from other values (p-value < 0.05). We removed outliers and reanalyzed data as needed until no outliers were detected. ANCOVA was performed to detect geographical differences of BMD and body composition traits. In this study, we choose age, weight, height, smoking^32,45,46^, alcohol use^47^, and regular physical exercise^33,48^ as covariates in the ANCOVA model. The comparison of percentage of regular physical exercise, alcohol use, and smoking were performed by Chi-square test. All other basic characteristics in this study were compared using ANOVA. Eta-squared based on ANOVA was also reported to reflect the effect size of differences due to its importance to empirical studies^34^. Given a sufficiently large sample size, a non-null statistical comparison will always show a statistically significant result. Therefore, in this study, we used both p-value and effect size as criteria to define statistical significance. The effect sizes were defined as small (η^2^ = 0.01), medium (η^2^ = 0.06), and large (η^2^ = 0.14) based on benchmarks suggested by Cohen^31^. A test is significant if η^2^ > 0.01 and p-values < 0.05.

### Brief summary

There is lack of studies on geographical variation influencing bone mineral density (BMD), obesity, and sarcopenia related traits in the US population. By comparing three study cohorts of various latitudes, we found no geographical effects on BMD. Obesity and sarcopenia related traits were significantly different among the three study cohorts.
